# Shared reading and independent book engagement in early childhood: associations with the home literacy environment in a Turkish pediatric outpatient population

**DOI:** 10.3389/fped.2026.1939681

**Published:** 2026-10-01

**Authors:** Enes Kaan Kilic, Furkan Kalayci, Kaan Çelebier, Aybars Ataus, Metehan Yaşar Tekin, Arif İsmet Çatak, Metin Yigit

**Affiliations:** 1 Department of Pediatrics, Ankara Bilkent City Hospital, Bilkent, Ankara, Türkiye; 2 Department of Pediatrics, Tokat Gaziosmanpasa University, Tokat, Türkiye; 3 Department of Pediatrics, Yildirim Beyazit University, Ankara Bilkent City Hospital, Bilkent, Ankara, Türkiye

**Keywords:** child literacy, childhood reading, early childhood, home literacy environment, shared reading

## Abstract

**Objective:**

To explore parental reading practices and factors associated with parental reading to children and children's independent engagement with books within the Home Literacy Environment (HLE).

**Methods:**

This cross-sectional study included 362 parents of children aged 1–6 years who attended the Pediatric Outpatient Clinic of Ankara Bilkent City Hospital between October and December 2025. Parents completed a 28-item questionnaire assessing their demographic characteristics, home literacy practices, parental reading habits, access to books, and children's independent book engagement. Firth's bias-reduced penalized likelihood logistic regression was used for multivariable analyses.

**Results:**

Shared reading was common, occurring daily in 47.3% and 2–3 times per week in 29.3% of families, most often during the evening or bedtime routines (64.2%). Maternal education was strongly associated with parental reading to the child; mothers with a university degree or higher had higher odds of reading to their children than those with a high school education or below [odds ratio (OR) = 14.31; 95% confidence interval (CI): 4.73–50.21; *p* < 0.001]. Independent book engagement was associated with older child age (OR = 1.38 per year; 95% CI: 1.11–1.73, *p* = 0.004), parental reading (OR = 3.82; 95% CI: 1.50–9.79,*p* = 0.005), a home library (OR = 2.48; 95% CI: 1.18–5.23,*p* = 0.017), and at least one parent with a regular reading habit (OR = 2.35; 95% CI: 1.02–5.18,*p* = 0.044), whereas maternal education was not independently associated with this outcome (*p* = 0.121).

**Conclusion:**

Parental reading and children's independent book engagement were associated with different components of the HLE. These exploratory findings suggest that both parental characteristics and everyday home literacy practices may be relevant to shared reading and independent book engagement, and may help inform future efforts to integrate family-centered literacy guidance into pediatric care.

## Introduction

Early childhood is a critical period in which environmental experiences contribute to cognitive, social, emotional, and language development (1, 2). Shared book reading is one of the most accessible parent–child activities during this period and has been associated with early language and literacy development (3, 4). Reflecting this evidence, the World Health Organization incorporates book-related interactions into its responsive caregiving framework, while the American Academy of Pediatrics (AAP) recommends promoting shared reading and early literacy from infancy as part of routine pediatric care (5, 6).

The Home Literacy Environment (HLE) provides a broader framework for understanding children's early literacy experiences and encompasses both access to print materials at home and parental practices, such as shared reading, parental modeling of reading behavior, and engagement with books. Within the classical Home Literacy Model proposed by Sénéchal and LeFevre (7), home literacy practices are broadly classified as informal and formal activities. Informal activities, particularly shared book reading, are centered primarily on the meaning of stories, whereas formal activities involve more explicit, code-focused practices, such as teaching letters, words, and writing. Given the developmental focus of the present study, which included children aged 1–6 years and examined shared reading, parental reading behaviors, access to books, and children's spontaneous engagement with books, the study primarily addressed the informal dimension of the HLE.

Within the informal dimension of the HLE, shared book reading has been consistently associated with children's language and literacy development (8–10), including vocabulary development and later reading comprehension. Shared reading has also been associated with later reading motivation and children's independent reading, suggesting that its relevance may extend beyond early language skills (11, 12).

Previous research has consistently associated characteristics of the HLE with children's early language and literacy development. Parental education and socioeconomic circumstances, parents' reading habits, access to books, and family literacy routines have all been linked to variations in children's literacy experiences. Parental modeling of reading, the availability of books and other literacy materials at home, and the incorporation of shared reading into everyday family routines have also been associated with greater child engagement in literacy-related activities (13–15). In a longitudinal study of German children, Niklas and Schneider (16) found that HLE was associated with early vocabulary and phonological awareness and partly mediated the associations between socioeconomic background and early literacy precursors. Similarly, using longitudinal Australian data, Kalb and van Ours (17) reported persistent associations between parental reading frequency and children's later reading and cognitive skills, with frequent reading corresponding to differences in reading performance approaching approximately 1 year of age-related development. Together, these findings highlight the importance of considering multiple dimensions of the home literacy environment rather than solely on shared-reading frequency.

Research in Türkiye has increasingly addressed HLE and related literacy practices. Altun (18) examined the relationship between preschool children's reading attitudes and their home literacy environments, while Altun et al. (19) subsequently evaluated home and classroom literacy environments in relation to vocabulary, phonological awareness, and print-related skills in a two-wave preschool study. More recently, Ergül et al. (20) situated research on HLE and literacy acquisition in Turkish within the broader cross-linguistic HLE literature, and Celik et al. (21) examined parental shared-reading practices and barriers in a Turkish pediatric clinical population. However, comparatively little is known about how multiple HLE components—including parental characteristics, reading habits, access to books, and family reading routines—are jointly associated with both parental shared reading and children's independent engagement with books within pediatric outpatient populations.

Accordingly, this cross-sectional study aimed to characterize parental reading attitudes, practices, and perceived barriers among parents of children aged 1–6 years attending an urban tertiary pediatric outpatient clinic in Türkiye, and to examine HLE characteristics associated with parental shared reading and children's independent engagement with books. Based on previous evidence linking parental education, parental reading practices, and access to books at home with different dimensions of the HLE and children's literacy experiences (13, 22, 23), we hypothesized that higher parental educational attainment, regular parental reading habits, and greater access to books at home would be associated with both outcomes.

## Methods

### Study design and participants

This cross-sectional study was conducted between October and December 2025 among parents who attended the Pediatric Outpatient Clinic of Ankara Bilkent City Hospital. The study population consisted of parents with at least one child aged 1–6 years. A total of 380 parents were initially recruited; after the exclusion of 18 participants with incomplete questionnaires, data from 362 participants were included in the final analysis. The mean maternal age was 35.6 ± 5.2 years (range: 24–63 years; *n* = 360), and the mean paternal age was 38.7 ± 5.6 years (range: 27–67 years; *n* = 360). Of the children, 51.4% were girls (*n* = 186) and 48.6% were boys (*n* = 176), with a mean age of 4.8 ± 2.4 years. Participation was voluntary, and all participants were informed of the purpose of the study and the confidentiality of the collected data. A convenience sampling approach was used, and all eligible parents attending the clinic during the study period were invited to participate.

### Questionnaire and data collection

Parents with at least one child aged 1–6 years, who were literate, and who could read and understand Turkish were included. Parents of children with special needs, developmental delays, or motor or intellectual disabilities for any reason were excluded.

Data were collected using a 28-item structured questionnaire developed by the research team to achieve the objectives of the present study. Although validated Turkish instruments are available for assessing the home early literacy environment (24, 25), this study focused on discrete and clinically interpretable reading practices rather than generating a composite HLE score. The questionnaire assessed demographic characteristics, selected components of the home literacy environment, and reading behaviors, including shared-reading frequency and duration, age at initiation, parental reading habits, access to books, family reading routines, perceived barriers, and children's independent engagement with books.

The questionnaire items were developed by the study team and were not adapted from previously published instruments. Before the main study, the questionnaire was reviewed by three pediatric specialists for clarity and content relevance and pilot-tested by 20 parents for comprehensibility and feasibility. Because the questionnaire assessed multiple distinct behaviors and characteristics rather than a single latent construct, no composite HLE score was calculated, and internal consistency across all 28 items was not assessed.

The questionnaire primarily assesses the informal dimension of HLE through several related but distinct constructs. Shared reading refers to parent–child book reading and is characterized by its frequency, duration, timing, and the person involved in reading. Children's independent book engagement refers to spontaneous interactions with books without parental assistance, including looking at pictures or retelling a familiar story in their own way. Parental reading habits reflected parents' self-reported regular engagement in reading, while reading in the child's presence was considered an indicator of reading-related parental modeling. Access to books was assessed through the availability and number of children's books and the presence of a children's bookshelf in the home. Family reading routines were assessed by asking whether the family had shared-reading time at home. These variables were treated as related components of HLE rather than as indicators of a single latent construct.

For the analysis, the selected questionnaire variables were dichotomized. For children's independent engagement with books and maternal and paternal regular reading habits, “Yes” responses were coded as positive, whereas “No” and “Rarely” responses were combined as negative. The derived variable “at least one parent with a regular reading habit” was coded as positive when either the mother or father was reported to read regularly. Parental reading to the child, family reading time at home, and the presence of a home library were recorded directly as binary yes/no responses. Parental education was categorized as high school or below versus university or higher for the regression analyses. The questionnaire was self-administered in the outpatient waiting area and required approximately 8–10 min to complete.

### Statistical analysis

Statistical analyses were performed using IBM SPSS Statistics version 25 (IBM Corp., Armonk, NY, USA) for descriptive and bivariate analysis. Descriptive statistics were expressed as numbers (percentages) for categorical variables and mean ± standard deviation for continuous variables. Associations between categorical variables were assessed using the chi-square test or Fisher's exact test, as appropriate. Given the limited number of non-events, parsimonious multivariable models were constructed to reduce the risk of overfitting and sparse data bias. Covariates were selected based on the study hypotheses and conceptual relevance to the home literacy environment while avoiding the inclusion of closely overlapping variables. Household income relative to expenses was also examined in sensitivity analyses but was not retained because it was not independently associated with either outcome after adjustment. Child age was included as a continuous covariate in both the models. Firth's bias-reduced penalized likelihood logistic regression was used for both the outcomes. Odds ratios (ORs), profile penalized likelihood 95% confidence intervals (CIs), and corresponding *p*-values were reported. A *p*-value <0.05 was considered statistically significant.

### Ethics statement

Ethical approval was obtained from the Ankara Bilkent City Hospital Clinical Research Ethics Committee (No: 1-25-1823). Written informed consent was obtained from all participants, and the study was conducted in accordance with the principles of the Declaration of Helsinki.

## Results

The majority of participants reported that they read books to their children (92.5%; *n* = 335). Reading frequency was most commonly daily (47.3%; *n* = 172) or 2–3 days per week (29.3%; *n* = 106), and the most common daily reading duration was 10–20 min (61.8%; *n* = 224). Shared reading was initiated within the first 3 years of life in 75.8% of children (*n* = 275), whereas 37.9% of families (*n* = 137) reported a family reading time at home. The most frequently reported difficulties in shared reading were lack of time (34.7%; *n* = 126) and difficulty sustaining the child's interest (29.1%; *n* = 106). The detailed demographic and behavioral findings are presented in Table 1.

**Table 1:** Demographic characteristics of participants and book-reading behaviors (*n* = 362).

Variable	Category	*n*	%
Child's sex	Girl	186	51.4
	Boy	176	48.6
Presence of siblings	Yes	203	56.1
	No	159	43.9
Preschool attendance	Yes	296	81.8
	No	66	18.2
Maternal employment status	Employed	203	56.0
	Not employed	159	44.0
Paternal employment status	Employed	332	91.7
	Not employed	30	8.3
Household income relative to expenses	Lower than expenses	62	17.1
	Approximately equal to expenses	208	57.5
	Higher than expenses	92	25.4
Parental reading to the child	Yes	335	92.5
	No	27	7.5
A person who reads	Mother only	193	53.2
	Both parents	104	28.7
	Father only	34	9.5
	Sibling	17	4.8
	Caregiver/teacher	14	3.8
Reading frequency	Daily	172	47.3
	2–3 days per week	106	29.3
	Once per week	42	11.7
	Rarely/never	42	11.7
Daily reading duration	None	11	3.2
	<10 min	50	13.8
	10–20 min	224	61.8
	20–40 min	69	19.0
	40–60 min	6	1.7
	>1 h	2	0.7
Age at initiation of reading	0–12 months	125	34.6
	1–3 years	150	41.2
	>3 years	87	24.2
Age at acquisition of first book	0–1 year	68	18.6
	1–3 years	170	46.7
	>3 years	124	34.4
Family reading time at home	Yes	137	37.9
	No	225	62.1
Time of reading	Evening/bedtime	233	64.2
	During the day	91	25.2
	Other	38	10.6
Book selection criteria^a^	Age-appropriateness and illustrations	286	78.9
	Message/theme of the book	191	52.6
	Child's interest	168	46.3
Purpose of reading^a^	Language development	208	57.2
	Quality time together	152	41.8
	Entertainment/routine	93	25.6
Reported difficulties^a^	Lack of time	126	34.7
	Difficulty sustaining the child's interest	106	29.1
	Limited variety of books	46	12.6

a For book selection criteria, purpose of reading, and reported difficulties, participants could select more than one response; therefore, percentages do not sum to 100%.

Parental reading to the child was significantly more frequent among children with higher maternal [*χ*^2^(1) = 50.94, *p* < 0.001] and paternal [*χ*^2^(1) = 23.65, *p* < 0.001] education, among children without siblings [*χ*^2^(1) = 5.58, *p* = 0.018], in families with a home library [*χ*^2^(1) = 21.23, *p* < 0.001], when at least one parent was a regular reader [*χ*^2^(1) = 5.84, *p* = 0.016], when the mother was a regular reader [*χ*^2^(1) = 4.72, *p* = 0.030], and when parents read in the child's presence [*χ*^2^(1) = 6.13, *p* = 0.013]. Parental reading did not differ significantly by preschool attendance [*χ*^2^(1) = 1.16, *p* = 0.282], paternal regular reading status [*χ*^2^(1) = 0.16, *p* = 0.690], or the child's sex [*χ*^2^(4) = 3.27, *p* = 0.514] (Table 2). Household income relative to expenses was associated with parental reading in the univariable analysis [*χ*^2^(2) = 13.50, *p* = 0.001], whereas no significant association was observed between household income and children's independent book engagement [*χ*^2^(2) = 3.10, *p* = 0.210].

**Table 2:** Variables associated with parental reading to the child (*n* = 362).

Variable	Category	Not read (*n*, %)	Read (*n*, %)	*χ*^2^(df)	*p*-value
Child's sex	Boy	13 (7.4%)	163 (92.6%)	3.27 (4)	0.514
	Girl	14 (7.5%)	172 (92.5%)		
Maternal education level	High school or below	22 (24.7%)	67 (75.3%)	50.94 (1)	<0.001
	University or higher	5 (1.8%)	268 (98.2%)		
Paternal education level	High school or below	18 (18.6%)	79 (81.4%)	23.65 (1)	<0.001
	University or higher	9 (3.4%)	256 (96.6%)		
Presence of siblings	No	6 (3.8%)	153 (96.2%)	5.58 (1)	0.018
	Yes	21 (10.3%)	182 (89.7%)		
Preschool attendance	No	7 (10.6%)	59 (89.4%)	1.16 (1)	0.282
	Yes	20 (6.8%)	276 (93.2%)		
Home library	No	19 (17.0%)	93 (83.0%)	21.23 (1)	<0.001
	Yes	8 (3.2%)	242 (96.8%)		
Father reads regularly	No	13 (8.0%)	148 (92.0%)	0.16 (1)	0.690
	Yes	14 (6.9%)	187 (93.1%)		
Mother reads regularly	No	8 (14.5%)	47 (85.5%)	4.72 (1)	0.030
	Yes	19 (6.2%)	288 (93.8%)		
At least one parent reads regularly	No	7 (16.7%)	35 (83.3%)	5.84 (1)	0.016
	Yes	20 (6.2%)	300 (93.8%)		
Parent reads in the presence of the child	No	8 (16%)	42 (84%)	6.13 (1)	0.013
	Yes	19 (6.1%)	293 (93.9%)		

a Data are presented as *n* (%). Associations were assessed using the chi-square test or Fisher's exact test, as appropriate.

No significant association was found between maternal education and daily reading duration [*χ*^2^(15) = 23.28, *p* = 0.078]. The association with paternal education approached, but did not reach, the conventional threshold for statistical significance [*χ*^2^(15) = 24.83, *p* = 0.052] (Table 3).

**Table 3:** Comparison of daily reading duration according to maternal and paternal education levels.

Variable		How long do you read to your child per day?							
		None, *n* (%)	<10 min, *n* (%)	10–20 min, *n* (%)	20–40 min, *n* (%)	40–60 min, *n* (%)	>1 h, *n* (%)	*χ*^2^(df)	*p*-value
Paternal education level	Primary school	2 (6.9)	5 (17.2)	14 (48.3)	8 (27.6)	0 (0)	0 (0)	24.83 (15)	0.052
	High school	3 (4.4)	5 (7.4)	44 (64.7)	15 (22.1)	1 (1.5)	0 (0)		
	University	5 (2.4)	32 (15.4)	134 (64.4)	35 (16.8)	2 (1.0)	0 (0)		
	Master's degree	1 (1.8)	8 (14.0)	32 (56.1)	11 (19.3)	3 (5.3)	2 (3.5)		
Maternal education level	Primary school	1 (3.6)	4 (14.3)	17 (60.7)	6 (21.4)	0 (0)	0 (0)	23.28 (15)	0.078
	High school	5 (8.2)	6 (9.8)	35 (57.4)	15 (24.6)	0 (0)	0 (0)		
	University	5 (2.3)	33 (15.5)	131 (61.5)	39 (18.3)	5 (2.3)	0 (0)		
	Master's degree	0 (0)	7 (11.7)	41 (68.3)	9 (15.0)	1 (1.7)	2 (3.3)		

a Data are presented as *n* (%). Associations were assessed using the chi-square test or Fisher's exact test, as appropriate.

Children's independent book engagement was significantly more frequent among children whose parents read to them [*χ*^2^(1) = 30.43, *p* < 0.001], when at least one parent was a regular reader [*χ*^2^(1) = 12.13, *p* < 0.001], when there was a home library [*χ*^2^(1) = 19.62, *p* < 0.001], and among children with higher maternal [*χ*^2^(1) = 15.23, *p* < 0.001] and paternal [*χ*^2^(1) = 7.46, *p* = 0.006] education levels. No significant association was found with sibling presence [*χ*^2^(1) = 2.32, *p* = 0.128] or with both parents being regular readers [*χ*^2^(1) = 1.79, *p* = 0.181]. In the Firth logistic regression model evaluating children's independent engagement with books, older child age (OR = 1.38 per year; 95% CI: 1.11–1.73; *p* = 0.004), parental reading to the child (OR = 3.82; 95% CI: 1.50–9.79; *p* = 0.005), the presence of a home library (OR = 2.48; 95% CI: 1.18–5.23; *p* = 0.017), and having at least one parent with a regular reading habit (OR = 2.35; 95% CI: 1.02–5.18; *p* = 0.044) were independently associated with the outcomes. Maternal education was not independently associated with children's independent book engagement (OR = 1.91; 95% CI: 0.84–4.29; *p* = 0.121) (Table 4).

**Table 4:** Firth logistic regression analyses of factors associated with parental reading to the child and children's independent book engagement.

Outcome	Predictor	OR	95% CI	*p*
Parental reading to the child	Child age, per year	1.38	0.97–1.99	0.073
	Maternal education (university or higher vs. high school or below)	14.31	4.73–50.21	<0.001
	Presence of siblings	0.60	0.19–1.64	0.318
	Presence of a home library	1.70	0.60–5.05	0.312
	At least one parent has a regular reading habit	2.82	0.92–8.32	0.066
Children's independent book engagement	Child age, per year	1.38	1.11–1.73	0.004
	Maternal education (university or higher vs. high school or below)	1.91	0.84–4.29	0.121
	Parental reading to the child	3.82	1.50–9.79	0.005
	Presence of a home library	2.48	1.18–5.23	0.017
	At least one parent has a regular reading habit	2.35	1.02–5.18	0.044

a All models were estimated using Firth's bias-reduced penalized likelihood logistic regression. ORs, 95% CIs, and *p*-values were derived from profile penalized likelihood estimates.

In the Firth logistic regression model evaluating parental reading to children, maternal education was the only variable independently associated with the outcome. Mothers with a university degree or higher had substantially higher odds of reading to their children than those with a high school education or below (OR = 14.31; 95% CI: 4.73–50.21; *p* < 0.001). Child age, presence of siblings, presence of a home library, and having at least one parent with a regular reading habit were not independently associated with parental reading to children (Table 4). In sensitivity analyses, household income was not independently associated with either outcome after adjustment.

## Discussion

This study explored the associations between children's engagement with books in early childhood and multiple, partially independent components of the HLE. The findings suggest distinct patterns of association between parental reading to children and children's independent engagement with books. We hypothesized that higher parental educational attainment, regular parental reading habits, and greater access to books at home would be associated with both outcomes; however, this hypothesis was only partially supported. Maternal education was strongly associated with parental reading to the child, both bivariately and after adjustment, but was not independently associated with children's independent book engagement. Conversely, having at least one parent with a regular reading habit and the presence of a home library were independently associated with children's independent book engagement but not with parental reading to children after adjustment. Children's independent book engagement was additionally associated with older children and parental reading itself, suggesting that this outcome may be shaped more by proximal, everyday HLE components than by parental educational attainment. These findings support the view of the HLE as a multidimensional home context in which parent–child interaction and access to print materials are jointly but not uniformly associated with early literacy-related behaviors (7).

Among the variables examined, maternal education was most strongly associated with parental reading: mothers with a university degree or higher had substantially higher odds of reading to their children than those with a high school education or below. This is consistent with previous studies showing that parental education is associated with shared-reading practices, early literacy exposure, and book availability at home (7, 13), as well as with recent meta-analytic evidence from low- and middle-income countries indicating that the home reading environment strengthens as families' educational resources increase (15). More specifically, Khanolainen et al. reported that higher maternal education was associated with more frequent shared reading (22). In a large study of 98,464 children across 44 low- and middle-income countries, Jeong et al. found that both maternal and paternal education were associated with caregivers' support for learning. However, maternal education consistently showed stronger associations than paternal education and was also more strongly associated with the number of books available in the home (26). The particularly strong association observed in our sample may therefore be consistent with a broader pattern where maternal education is especially closely related to literacy-supportive characteristics of the home environment, although paternal education also contributes to these practices. This interpretation may also be considered in the context of national educational patterns. According to TÜİK, the proportion of higher-education graduates in the 25–34-year age group was 48.9% in 2024 (27), indicating that maternal educational attainment remains an important and variable family-level characteristic of the Turkish population. The attenuation of the income association after adjusting for maternal education may reflect the overlap between these socioeconomic indicators.

Beyond shared reading, our multivariable analysis identified four factors independently associated with children's self-initiated engagement with books: child age, presence of a home library, having at least one parent with a regular reading habit, and being read to by a parent. This pattern aligns with the view of HLE as a construct encompassing shared reading, parental modeling, and access to printed materials (7, 16). Among the identified factors, being read to by a parent was most strongly associated with the outcome, which is consistent with evidence linking shared reading to children's language- and literacy-related outcomes (3, 17). This finding suggests that direct adult–child reading interactions may go beyond simple book exposure and support children's transition from shared to more autonomous engagement with books. Access to books and parents' reading habits may complement this process by making books a more visible, familiar, and valued part of children's everyday environment.

Although most parents in our sample reported reading to their children, regular family reading routines were less common, and only a minority had initiated shared reading in the first year of life. This gap between reported reading and consistent practice is notable, particularly given the educational profile of the sample, and suggests that even in educationally advantaged urban Turkish families, the AAP recommendation to begin shared reading in infancy and embed it into routine pediatric primary care may not yet be consistently implemented in Türkiye. Previous data from Türkiye have also shown that the early initiation of shared reading remains limited (21). The relatively high overall reading rate observed here is consistent with the hospital-based tertiary-care setting and the high educational level of participating parents, which may limit the generalizability of these findings to broader Turkish populations. This finding may also be interpreted in the context of the broader reading culture in Türkiye. In the 2019 Türkiye Reading Culture Survey, 44.4% of respondents identified guidance for parents as an important strategy for promoting a reading culture, underscoring the perceived importance of the family context in supporting reading habits (28).

The most frequently reported barriers to shared reading were lack of time and difficulty sustaining the child's interest, suggesting that parental reading behavior is associated not only with attitudes or educational level but also with practical and relational factors in everyday family life. These patterns are consistent with a recent international meta-synthesis that identifies time constraints, parental fatigue, child engagement challenges, and limited access to materials as recurring barriers to shared reading across diverse family contexts (29). From a pediatric primary care perspective, these findings support the use of brief, structured prompts during well-child visits—for example, asking about evening routines or framing shared reading as a short daily practice—as low-burden strategies aligned with the AAP's emphasis on integrating evidence-based literacy promotion into routine pediatric care (6). The predominance of bedtime reading may reflect that evening hours provide one of the most consistent opportunities for parent–child interaction within the daily family routine. This finding also aligns with the American Academy of Pediatrics' “Brush, Book, Bed” approach, which incorporates shared reading into a simple and predictable bedtime routine for young children (30).

A gendered pattern was also evident in the shared-reading practices. Mothers were reported as the primary readers more frequently than fathers, although both parents were also involved in shared reading. Together with the strong association observed between maternal education and parental reading to the child, this distribution suggests that mothers played a particularly prominent role in shared-reading practices in our sample. When shared reading is addressed primarily through mothers, opportunities to engage fathers may be missed. Pediatric literacy promotion frameworks recommend engaging both parents during well-child visits (6), and our findings suggest that fathers may need to be more explicitly included in shared-reading discussions in similar clinical settings.

These findings have practical implications for early literacy promotion. Evidence suggests that book distribution alone may be insufficient to enhance the home literacy environment: a recent study found that the receipt of a book without accompanying anticipatory guidance and clinician modeling of shared reading was not associated with improved home literacy environments, whereas the combination of these components showed a dose–response relationship (31). This suggests that pediatric providers should be trained not only to distribute books but also to guide families toward developmentally and linguistically appropriate books and model and coach interactive shared-reading behaviors during well-child visits, consistent with AAP recommendations for structured literacy promotion (6). Programs implementing multiple components of guidance and modeling, such as Reach Out and Read, have shown consistent increases in caregiver reading frequency and shared-reading behaviors across repeated years of exposure (32). Beyond the clinical encounter, cross-sector partnerships between pediatric primary care, early childhood education, libraries, and community organizations can help reinforce consistent literacy messaging and extend population-level reach beyond single points of contact (6, 33). Hospitals and pediatric outpatient settings can further support these efforts by making developmentally appropriate books visible and accessible and by providing child-friendly environments that encourage book engagement.

Several additional family and child characteristics were associated with varying degrees of shared-reading practices. When selecting books, parents most commonly consider age-appropriateness and illustrations, followed by educational value and the child's interest. This is broadly consistent with previous work showing that parents' picture book preferences are related to the characteristics of the books themselves as well as to their perceptions of their children's preferences (34). Children with siblings were less likely to be read to in the bivariate analysis; however, sibling presence was not independently associated with parental reading after adjustment for the Firth regression model. The bivariate association may reflect competing demands for parental attention or differences in reading interactions within larger families, including the involvement of older siblings. Similar patterns of reduced individualized parent–child interactions with increasing family size have been reported previously (13). By contrast, preschool attendance was not significantly associated with parental reading, suggesting that preschool participation and shared-reading practices at home may represent partly distinct aspects of children's early literacy experiences.

This study has several methodological limitations. First, its cross-sectional design does not allow for causal inference. The temporal direction of the observed associations cannot be established, and reverse causation is also possible; for example, children who show greater spontaneous interest in books may encourage more frequent parental reading or greater acquisition of books within the home. Because both HLE characteristics and child book-engagement outcomes were reported concurrently by the same parent, the observed associations may be affected by common method bias. Parental self-report may also be subject to social desirability and recall biases. Some odds ratio estimates remained imprecise, as reflected by wide confidence intervals, particularly in the parental reading model with a limited number of non-events, and should therefore be interpreted with appropriate caution.

Second, the questionnaire was developed by the research team rather than using a previously validated HLE instrument, which may limit the direct comparability of our results with those of studies using standardized measures. Although the questionnaire underwent expert review and pilot testing for clarity, comprehensibility, and feasibility, its individual items were not formally evaluated for test–retest reliability; therefore, some degree of item-level measurement variability cannot be excluded. In addition, children's independent engagement with books was assessed using a single parent-reported item, rather than a validated multi-item measure. Third, the questionnaire addressed only the informal pathway of the Home Literacy Model (shared reading, home book access, and parental reading modeling) and did not capture formal literacy practices such as the direct teaching of letters, words, and writing, which may become increasingly relevant as children approach school age (7).

The sample also reflected the catchment population of a tertiary urban hospital. The proportion of university-educated mothers in our sample substantially exceeded the corresponding national figure for women aged 25–34 in Türkiye (48.9% in 2024) (35). The findings should therefore be interpreted as representing literate, urban, tertiary care-attending families and may not be generalizable to populations with lower educational attainment or to rural settings. Future studies using prospective designs, validated instruments, and broader sociodemographic sampling, including non-tertiary, lower-education, and rural populations, are needed to extend and further examine these findings in Turkish populations.

In conclusion, this study explored the multidimensional nature of the Home Literacy Environment in early childhood. Maternal education was strongly associated with parental reading to the child, whereas children's independent engagement with books was independently associated with older child age, parental reading to the child, the presence of a home library, and at least one parent with a regular reading habit. These findings suggest that children's early engagement with books reflects not only parental characteristics but also everyday literacy experiences within the home, including shared reading, parental modeling, and access to books. Pediatric primary care may provide an important setting for supporting these experiences through family-centered literacy guidance, beginning early in childhood.

## Data Availability

The raw data supporting the conclusions of this article will be made available by the authors without undue reservation.
