# Supplementary material for: Shared reading and independent book engagement in early childhood: associations with the home literacy environment in a Turkish pediatric outpatient population
Source: Front Pediatr. 2026 Oct 1;14:1939681. doi: 10.3389/fped.2026.1939681 (PMC13668856; doi:10.3389/fped.2026.1939681)
Supplement: Supplementary file 1 [file Datasheet1.pdf]

Supplementary File S1

English Translation of the Data Collection Form

Republic of Türkiye – Ministry of Health
Ankara Bilkent City Hospital
Scientific and Ethical Evaluation Board for Medical Research

DATA COLLECTION FORM

Form No: ____________________ Date: ____________________

1.

A) Mother's age: ____________________
B) Father's age: ____________________

2.

A) Child's age: ____________________

3. What is your child's sex?

☐ Female
☐ Male

4.

A) Number of siblings: ____________________
B) If applicable, ages of siblings:

☐ First sibling: ____________________
☐ Second sibling: ____________________
☐ Third sibling: ____________________

5. Does your child attend a preschool education institution (e.g., kindergarten, nursery/daycare)?

☐ Yes
☐ No

6.

A) How does your family's total monthly income compare with your monthly expenses?

☐ Income is lower than expenses
☐ Income is approximately equal to expenses
☐ Income is higher than expenses

B) Is the mother employed?

☐ Yes
☐ No

C) Is the father employed?

☐ Yes
☐ No

7.

A) What is the mother's educational level?

☐ Primary education or below
☐ High school
☐ University
☐ Master's degree or higher

B) What is the father's educational level?

☐ Primary education or below
☐ High school
☐ University
☐ Master's degree or higher

8.

A) Do you read books to your child?

☐ Yes
☐ No

B) If someone other than you provides care for your child (e.g., caregiver, grandparent), does that person read books together with your child?

☐ Yes
☐ No
☐ No caregiver other than the parent

9. Who most frequently reads books to your child?

☐ Mother
☐ Father
☐ Mother and father equally
☐ Sibling
☐ Caregiver other than the parents
☐ Teacher

10.

A) How often do you read books to your child?

☐ Every day
☐ 2–3 days per week
☐ Once a week
☐ Rarely
☐ Never

B) On days when you read to your child, approximately how long do you spend reading?

☐ Less than 10 minutes
☐ 10–20 minutes
☐ 20–40 minutes
☐ 40–60 minutes
☐ More than 1 hour
☐ I do not read to my child

11. How old was your child when you first started reading books to them or showing them picture cards?

☐ 0–6 months

☐ 6–12 months

☐ 12–24 months

☐ 24–36 months

☐ 36–48 months

☐ Older than 48 months

12. At what times of the day do you read books to your child? *(Select all that apply.)*

☐ Before sleep/bedtime

☐ After waking up

☐ During meals

☐ During playtime

☐ Other: ____________________

13. Does your family have a designated time when all family members read books together at home?

☐ Yes

☐ No

14. Approximately how many books does your child have?

☐ 1–5 books

☐ 5–10 books

☐ 10–20 books

☐ 20–30 books

☐ More than 30 books

☐ No books

15. When did you first buy a book or picture cards for your child?

☐ Before birth

☐ During the first 6 months

☐ 6–12 months

☐ 1–2 years

☐ 2–3 years

☐ 3–4 years

☐ Older than 4 years

☐ I have not bought any

16. Does your child engage with books independently, without your assistance (e.g., looking at pictures or retelling a familiar story in their own way)?

☐ Yes

☐ No

☐ Rarely

17. Do you read your own books in your child's presence?

☐ Yes
☐ No
☐ Rarely

18.

A) Does the mother regularly read books for herself?

☐ Yes
☐ No
☐ Rarely

B) Does the father regularly read books for himself?

☐ Yes
☐ No
☐ Rarely

C) If the child has siblings, do they regularly read books?

☐ Yes
☐ No
☐ Rarely
☐ No siblings

19. Do you read or review the books before giving them to your child?

☐ Yes
☐ No

20. Approximately how much time does your child spend in front of a screen (television, smartphone, or tablet) per day?

☐ Less than 15 minutes
☐ 15–30 minutes
☐ 30–60 minutes
☐ 1–2 hours
☐ More than 2 hours
☐ No screen exposure

21. What factors do you consider when selecting books for your child? *(Select all that apply.)*

☐ Topic and content
☐ Online reviews
☐ Recommendation from school/kindergarten
☐ Print and illustration quality
☐ Book length/thickness
☐ Suitability for the child's age and sex
☐ Recommendations from official institutions (e.g., Ministry of National Education)
☐ Foreign-language content
☐ Recommendations from parents of children of a similar age
☐ Interactive/activity-based content
☐ Other: ____________________

22. Do you buy books as gifts for your child?

☐ Yes

☐ No

23. How often do you go to buy books together with your child?

☐ Never

☐ Once a week

☐ 2–3 times a month

☐ Once a month

☐ 3–4 times a year

☐ Once a year

24. Why do you read books to your child or encourage your child to engage with books? *(Select all that apply.)*

☐ To improve vocabulary

☐ To stimulate imagination

☐ To spend enjoyable and quality time together

☐ To support the child's ability to express themselves

☐ To keep the child occupied

☐ To help the child fall asleep

☐ To facilitate feeding/mealtimes

☐ Other: ____________________

25. When choosing a book for your child, do you prefer it to contain a specific "lesson" or "message"?

☐ Yes

☐ No

26. Do you think there is sufficient variety of children's books available in Türkiye?

☐ Yes

☐ No

☐ No opinion

27. Is there a home library/bookcase in your home?

☐ Yes

☐ No

28. What difficulties do you encounter when reading books to your child or encouraging your child to engage with books? *(Select all that apply.)*

☐ The child becomes bored or sleepy

☐ The book does not attract the child's interest / the child does not like the book

☐ The parent has difficulty finding enough time

☐ Books are expensive

☐ Screens (smartphone/tablet/television) attract the child's attention more

☐ Toys attract the child's attention more

☐ Other: ____________________

Note: This document is an English translation of the original Turkish data collection form used in the study. Item numbering and response structure have been preserved.

Supplementary Table S1. Operational definitions and coding of principal questionnaire variables

The table below specifies the source questionnaire item, original response options, and analytical coding used for the principal variables included in the analyses.

Variable	Questionnaire item	Original response options	Analytical coding
Parental reading to the child	Q8A	Yes / No	Yes = 1; No = 0
Family shared reading time	Q13	Yes / No	Yes = 1; No = 0
Independent engagement with books	Q16	Yes / No / Rarely	Yes = 1; No or Rarely = 0
Maternal regular reading habit	Q18A	Yes / No / Rarely	Yes = 1; No or Rarely = 0
Paternal regular reading habit	Q18B	Yes / No / Rarely	Yes = 1; No or Rarely = 0
At least one parent with a regular reading habit	Derived from Q18A and Q18B	Derived variable	Yes = 1 if either parent was coded 1; otherwise = 0
Perceived limited variety of children's books	Q26	Yes / No / No opinion	No = perceived limited variety
Home library	Q27	Yes / No	Yes = 1; No = 0

Note. For Q16, Q18A, and Q18B, responses of "Rarely" were grouped with "No" for dichotomized analyses. "At least one parent with a regular reading habit" was coded as positive when either the mother or the father was reported to read regularly.
